# Editorial: “Promoting a lifespan approach to oral health and oral health-related quality of life”

**DOI:** 10.3389/froh.2026.1942706

**Published:** 2026-08-04

**Authors:** Mark R. Luborsky, Kitty Jieyi Chen, Praveen Hoogar, Judith Jones

**Affiliations:** 1 Wayne State University, Detroit, MI, United States; 2 Sun Yat-Sen University Guanghua School of Stomatology, Guangzhou, China; 3 School of Social Sciences, The Apollo University, Chittoor, India; 4 University of Detroit Mercy, Detroit, MI, United States

**Keywords:** biocultural anthropology, life-course analysis, oral healh, public health, social determinansts of health, health equity, oral health-related quality of life, life-course approach

Oral health unfolds across time and place, shaped by contextual and temporal forces that remain inadequately captured in conventional clinical models. A life-course approach holds that health at any given stage reflects the cumulative influence of biological, behavioral, and social exposures across earlier stages, from gestation through old age, rather than a state determined solely by present circumstances. This Research Topic was assembled to move beyond the individual mouth and consider oral health as it is embedded in life-course trajectories, socioeconomic circumstances, and policy environments. The rationale is not merely descriptive. Poor oral health is tied to systemic chronic disease, adverse pregnancy outcomes, and cognitive decline in later life, and it shapes nutrition, social participation, and self-esteem in ways that extend well beyond the dental chair. The contributions gathered here span childhood through old age and range from community-based qualitative work to large-scale registry analysis, and together they probe not only where oral health disparities arise but where meaningful intervention becomes possible.

## Life-course contexts of oral health

### Adolescent and early-life oral health

Oral health trajectories are most malleable in childhood, and the costs of early neglect compound over a lifetime. Kirby et al. (2025) demonstrate this directly through the Great Beginnings for Healthy Native Smiles program, in which Community Health Representatives from two partnering Indigenous communities delivered a culturally tailored motivational interviewing intervention to 41 caregivers of young children. Twenty-two of the 23 caregivers who completed debriefing interviews reported at least one change in at-home oral health behavior, and the authors attribute this success to trust built among community members rather than to the content of the education alone. The structural dimension of childhood oral health is reinforced by Adeagbo et al. (2025), whose photovoice study with 38 caregivers of children with orofacial clefts at a Nigerian teaching hospital found that feeding difficulty, driven largely by nasal regurgitation, and the cost of specialized formula and feeding equipment were the two dominant burdens, and that caregivers relied heavily on faith and spirituality rather than formal support systems.

Moving into early adulthood, Coll Campayo et al. (2026) compare students across dentistry, nutrition, and fine arts programs in Spain and find that lifestyle and oral health habits diverge sharply by field of study, with dentistry students reporting the lowest rate of recent oral pain and fine arts students the highest, situating oral health behavior within educational socialization rather than treating it as fixed at adolescence. Complementing this, Balgiu et al. (2025) surveyed 775 young Romanian adults and modeled dental self-confidence, self-esteem, and subjective well-being: self-esteem partially mediated the relationship between dental confidence and well-being, with the two constructs together accounting for nearly 60% of the variance in well-being.

The clinical timing of care itself also carries psychological weight. Li et al. (2025) surveyed 450 adolescent orthodontic patients in Chengde, China, and found that 32% exhibited depressive symptoms on the Beck Depression Inventory for Youth, a rate well above general adolescent prevalence. The risk was concentrated among those with the longest treatment delays: patients waiting 181–360 days for care faced more than four times the odds of depressive symptoms relative to those treated promptly, even after adjusting for demographic factors and malocclusion severity. Whereas Coll Campayo et al. and Balgiu et al. locate psychological well-being in habits and self-perception, this finding locates it partly in health-system responsiveness itself, positioning delayed access to routine care as a determinant of adolescent mental health in its own right rather than a downstream inconvenience.

### Adult oral health and systemic correlations

For adults, the contributions in this collection focus on chronicity and the connections between oral and systemic disease. Madfa et al. (2026) tested an AI-based chatbot as a pre-treatment intervention among adult dental patients in Saudi Arabia and found statistically significant reductions in anticipatory dental anxiety across all five procedure scenarios tested, with the largest reductions for tooth drilling and local anesthetic injection. However, the authors note that without a control group, the design cannot rule out attention or habituation effects. Weng et al. (2025) extend the logic of mind–body integration to a chronic, non-periodontal condition, surveying 358 patients with recurrent aphthous stomatitis and finding that prolonged ulcer duration and a preference for Traditional Chinese Medicine interventions independently predicted both anxiety and depression, while dietary factors cut in different directions: frequent fried-food consumption worsened both anxiety and depression, whereas frequent vegetable intake was protective against both. Read together, these two papers suggest that the psychological experience of oral disease is not a peripheral concern but a determinant of whether and how systemic risk is managed.

### Oral health in older adults

Aging introduces vulnerabilities with no clear analog earlier in the life-course. Caetano-Santos et al. (2026), in a cross-sectional study of older adults in Portugal, identify the socioeconomic and self-perception factors most predictive of oral health behaviors in this population, extending a literature the authors note has been dominated by non-European samples.

A recurring finding across the geriatric contributions is that outcomes in later life are predicted less by chronological age than by cumulative exposure across earlier decades. Luborsky et al. (2026) illustrate this through a community-based oral health equity project with older, disadvantaged, predominantly minority residents of inner-city Detroit, using qualitative interviewing to unpack how participants construct their own oral health self-ratings and beliefs, information the authors argue is essential for providers and policymakers precisely because so little is currently understood about how these appraisals form. Yang et al. (2025) extend this life-course logic to a specific clinical event, surveying 298 older adult stroke patients and modeling, through structural equation analysis, how oral health literacy relates to oral frailty through parallel mediating pathways involving oral health self-efficacy and oral health-related quality of life — with higher literacy associated with lower frailty largely by way of the confidence and habits patients then bring to their own care. Together, these two studies argue for interventions calibrated to accumulated exposure and self-efficacy rather than to age as a proxy.

Contexts across time and space: social determinants and disparities.

A second cluster of contributions turns from the individual life-course to the structural and geographic contexts in which oral health is produced. Advances in clinical materials continue on their own track, regardless of these structural questions: Roma et al. (2026) review three decades of development in endodontic bioceramics, from mineral trioxide aggregate through newer materials such as Biodentine and BioAggregate, concluding that while individual-level restorative outcomes have improved substantially, controlled clinical evidence for many newer products still lags behind laboratory promise. The juxtaposition is instructive: technical progress at the level of the individual tooth proceeds on a timeline largely independent of the structural and economic questions raised elsewhere in this collection.

Geography and environment shape oral health independently of individual choice. Okore et al. (2026) surveyed 52 households in Nakuru County, Kenya, where geogenic fluoride contaminates groundwater, and found that most residents correctly understood fluoride and fluorosis, with 81% and 90% respectively. Yet the majority still reported doing nothing about it: 67% expressed indifference toward their exposure, and 65% relied on ineffective practices such as boiling water or aggressive brushing rather than genuinely protective measures. The gap the authors identify is not one of knowledge but of resources, infrastructure, and trusted alternatives. Infrastructure carries similar weight near the clinic itself: Vatanparast et al. (2025) tested dental unit waterlines and clinic taps at rural and urban clinics in Saskatchewan, Canada, over eight months and found that rural dental unit waterlines failed bacterial safety standards at more than double the rate of the urban clinic (36.4% vs. 14.9%), while nearly half of rural tap-water samples failed outright, with zero failures in the urban clinic — and that rural clinics were also markedly slower to retest and remediate. The disparity here is not about patient behavior at all, but about the infrastructure that delivers care in the first place.

Dental public health has historically relied on individual behavior change as its primary lever, a strategy these contributions suggest is insufficient on its own. Mendes et al. (2025) address this by developing a new multidimensional oral health indicator for 1,034 first-time patients at a Portuguese university dental clinic, combining a validated oral health values scale, the OHIP-14 quality-of-life measure, and clinical caries data into a single composite score. Active smoking (odds ratio 3.12) and lower educational attainment (odds ratio up to 2.94) emerged as the strongest predictors of a degraded oral health profile, ahead of age or self-reported periodontitis — providing clinicians and policymakers with a single tool that captures both subjective and clinical burden rather than as separate figures.

### Policy initiatives and implementation

The policy-oriented contributions in this Research Topic share a common preoccupation: the distance between what is known to work and what is funded, implemented, and sustained. Northridge and Lieberman (2025) report the capstone findings of a five-year postdoctoral dental residency program built around more than 110 community health centers across 30 US states, Puerto Rico, and the US Virgin Islands, training dental faculty and residents in shared decision-making and motivational interviewing and testing interprofessional pilot projects on social-determinant screening, HPV vaccination referral, telehealth, and sugary-drink education. By 2025, 114 program graduates, 12% of the network's active dental faculty, were themselves practicing at community health centers, and simple workflow changes, such as flagging overdue dental visits at medical wellness checks, doubled monthly referrals to dentistry. Their account argues that closing the mouth–body divide in everyday clinical workflow may do as much for equity as dental-specific policy reform pursued on its own.

The scale of the underlying access problem is documented empirically by Yu (2025), whose analysis of All of Us data on nearly 128,000 adults finds that social determinants of health, income, insurance status, and housing instability among them are consistently associated with dental care access across the adult life-course, more so than physical or mental health challenges considered in isolation. That the same structural predictors, income, insurance, and housing, recur across a 128,000-person national dataset and a 52-household qualitative study in rural Kenya is itself a finding: the mechanisms linking social position to oral health appear stable across method and setting, even where their expression differs.

For policymakers, the practical implication is that oral health strategies are likely to have the greatest reach when anchored to touchpoints patients already have with the health and social systems pediatric well-child visits, insurance and Medicaid enrollment, primary care check-ins in mid-life, and home- or facility-based care in old age rather than designed as freestanding, age-specific programs.

## Conclusion

This Research Topic brings together contributions from dental research, public health, health psychology, and the social sciences to situate oral health within the human life-course rather than within the boundaries of a single clinical specialty. Read as a set, the sixteen articles converge on three observations that recur across otherwise disparate methods and populations. First, oral health trajectories are set early and carried forward: outcomes in adulthood and old age, from Detroit to rural Kenya to Portuguese dental clinics, reflect accumulated exposure rather than an isolated present-day clinical state. Second, the psychological and social dimensions of oral health, including confidence, anxiety, self-rated status, and quality of life, are not secondary to biomedical outcomes but appear to mediate them, in some models accounting for nearly 60% of the variance in a downstream outcome. Third, the persistent separation of oral health from general medical and public health systems, evident in financing, infrastructure, and workforce alike, is a structural problem rather than a knowledge gap: several contributions show that people already know what protects their oral health yet lack the resources, coverage, or systems to act on it. Contributions that test integration directly, whether through primary care embedding, composite indicators, or community-based training, offer the clearest evidence of what closing that gap might achieve. The questions that remain, about causal mechanisms, the timing of intervention, and the translation of evidence into durable policy, are unlikely to be resolved by any one discipline working in isolation, and we hope this collection makes the case for continuing that work across disciplinary lines.

Future research would benefit from longitudinal designs capable of tracking these accumulating exposures directly, implementation science evaluating how integration strategies such as those reported here scale beyond single institutions, and sustained interdisciplinary collaboration to evaluate preventive interventions across the life-course.

